# “It’s no use saying it in English”: A qualitative study exploring community leaders’ perceptions of the challenges and opportunities with translating and interpreting COVID-19 related public health messaging to reach ethnic minorities in Australia

**DOI:** 10.1371/journal.pone.0284000

**Published:** 2024-02-29

**Authors:** Holly Seale, Ben Harris-Roxas, Anita E. Heywood, Ikram Abdi, Abela Mahimbo, Lisa Woodland, Emily Waller

**Affiliations:** 1 School of Population Health, Faculty of Medicine and Health, University of New South Wales, Sydney, New South Wales (NSW), Australia; 2 National Centre for Immunisation Research and Surveillance, Westmead, NSW, Australia; 3 The Children’s Hospital at Westmead Clinical School, Faculty of Medicine and Health, University of Sydney, Sydney, NSW, Australia; 4 School of Public Health, Faculty of Health, University of Technology, Sydney, NSW, Australia; 5 NSW Multicultural Health Communication Service, South Eastern Sydney Local Health District, NSW Health, Sydney, NSW, Australia; 6 Centre for Primary Health Care and Equity, University of New South Wales, Sydney, NSW, Australia; Caleb University, NIGERIA

## Abstract

**Background:**

The Australian Government implemented a range of public health response strategies and communication approaches to reduce the spread of COVID-19; however, concerns have been raised around a failure to sufficiently consider culturally and linguistically diverse (CaLD) communities in these processes. This research aimed to understand the factors that have impacted COVID-19 communication and engagement efforts during the pandemic from the perspective of key CaLD community and faith-based leaders. A further aim was to understand the processes that could be adopted to support future communication strategies, including promoting pandemic-related vaccines.

**Approach:**

This study included 29 key informant interviews with community and faith-based leaders in New South Wales, Australia.

**Results:**

The overwhelming message from community leaders was a sense of shared responsibility between their organisations and governments in communicating pertinent and accurate COVID-19 related information to CaLD communities. They expressed a sense of duty to keep their community members safe. However, community leaders and others shouldered significant costs related to resources and time that need to be acknowledged by governments in preparing for future disease outbreaks. They felt that governments should consider: 1) improving communication between governments and CaLD organisations; 2) responding to the specific CaLD needs with greater agility; 3) foregrounding social media in their communication strategy; 4) reinvesting in local public health units to know their population; 5) developing a health ambassadors model program; 6) preparing a hybrid model of translators/interpreters to fill the gap; and, 7) reimagining vaccine information campaigns to target CaLD communities better.

**Conclusion:**

Given the technical details about the COVID-19 virus conveyed in government information campaigns and the media, ensuring the most vulnerable populations, including people from CaLD backgrounds, access clear, concise and timely public health messaging from governments and community organisations requires further attention.

## Introduction

Much has been written about the global COVID-19 pandemic’s impact on vulnerable communities, mainly ethnic minority populations (the convention in Australia is to refer to such groups as culturally and linguistically diverse (CaLD) populations. Thus, we will be using this term throughout the paper) [1–5]. Numerous studies have documented the heightened risk from SARS-CoV-2 and the disparity in COVID-19 cases and deaths amongst a diverse group of migrants, refugees, asylum seekers and other ethnic groups [6, 7]. Evidence suggests that in some settings, these at-risk groups of migrants have been excluded from the national response [2, 5]. Several factors have contributed to this, including a lack of consideration and tailoring given to the needs of CaLD population groups within pandemic response strategies and inaction amongst governments to ensure that resources are available in a wide range of languages and culturally relevant [6]. Reviews focused on the quality of COVID-19 communication materials have highlighted a lack of resources on disease prevention for some migrant population groups (people in refugee camps or informal settlements), limitations in the number of languages information was translated into, issues with the complexity of information (readability, understandability, actionability), and with the quality of the translations [2, 8, 9]. Studies from Denmark, Australia, Canada, and the US have documented delays in the availability of official guidance in relevant languages for the respective countries and issues with the approaches used to disseminate resources into CaLD communities, with a particular focus on groups with lower language and literacy levels, or communities with oral languages [3, 10, 11]. These issues are heightened amongst older people from CaLD backgrounds, which can be attributed to their inability to search for health information online and a communication gap between adults from CaLD backgrounds and the public health information dissemination system [12]. People from CaLD backgrounds can be left behind in their access to and understanding of recommendations, compounded by cultural discordance and mistrust of health institutions [13].

In Australia, English is the dominant language; however, many people speak a language other than English within their families and communities [14]. There are over 200 languages spoken in Australia, including over 50 actively spoken Australian Indigenous languages. The availability of information in other languages often depends on the size of the community, the level of community infrastructure, and the engagement of the community with health services around other health-related issues. In Australia, speakers of languages with sizeable numbers of practitioners (e.g. Mandarin, Arabic, Vietnamese) may glean information from national- or community-based radio stations and newspapers, among other modes of communication. However, other communities are classified as linguistic minorities, which do not have this level of community infrastructure [15]. Research has found that people from CaLD communities identify strategic stakeholders (referred to in this paper as ‘information intermediaries’) for disseminating information within groups [16, 17]. Information intermediaries could include staff from community organisations, community or religious leaders, bilingual case or youth workers or ‘natural’ leaders (for example, a person who has completed medical training but does not practice in Australia) across the age spectrum. Through influencing the flow of communication, information intermediaries shape and inform their community’s reality and knowledge in a culturally appropriate and salient manner [18].

While guidelines and reports highlight the crucial role of these information intermediaries in supporting communities during the COVID-19 pandemic, less is known about the experiences of these information intermediaries in terms of their perceptions of the COVID-19 response, the needs of their communities and their experiences in ‘bridging the gap’ between levels of government and the community in communicating pertinent COVID-19 information. Given the concerns about the Australian Government insufficiently considering the needs of CaLD communities in their public health response strategies and communication approaches to reduce the spread of COVID-19 [19, 20], this research aimed to understand the factors that have impacted COVID-19 communication and engagement efforts during the pandemic from the perspective of key CaLD community and religious leaders. A further aim was to understand the processes that could be adopted to support future communication strategies, including promoting pandemic-related vaccines, as proposed by CaLD information intermediaries.

## Methods

### Study design and study population

A qualitative study was designed using semi-structured, in-depth phone interviews with people who self-identified as community or faith-based leaders. The focus was on CaLD communities, including people born in mainly non-English-speaking countries where English is not the primary language spoken at home [16].

Key organisations were identified, including migrant resource centers, refugee health services, settlement services, advocacy groups, community-based organisations, and government organisations, to promote the study opportunity to known leaders within their networks. Information, including key study details, the researchers conducting the study, funders, inclusion criteria, participant involvement, data confidentiality, and withdrawal details, was sent out via these networks, with potential participants encouraged to connect with the research team for further information or to express interest. Recruitment targeted those living within New South Wales (NSW), Australia. See S1 Appendix for more details outlined in the COREQ consolidated criteria for reporting qualitative research.

### Ethics approval

The Human Research Ethics Advisory Panel at the University of New South Wales reviewed and approved this study (HC200776). Aligned with this approval and given varying literacy levels among key informants, informed verbal consent was collected from all participants and recorded at the start of the interview. Participants were only included in the study when full verbal consent had been received, and participants were informed they could withdraw at any time. This study did not collect any identifiable personal information from the participants.

### Data collection and analysis

The researchers developed and reviewed an interview guide to identify critical areas of interest for the study. The questions related to the following topics: perspectives towards the current communication approach being used by the government, factors affecting communication and engagement with CaLD communities, the communication roles and influences of different agencies, and suggested options that could be adopted to enhance communication and engagement around the COVID-19 vaccine program. Questions were asked in an open-ended manner to allow room for expansion [21]. Two pilot interviews were conducted to test and refine the interview guide before undertaking the recruitment strategy. Minor changes to the interview guide were made to improve the flow and order of the questions and add supplementary probing questions. Interviews lasted approximately 30–40.

Minutes and data were collected between March and September 2021. Purposeful sampling was used to capture and describe various experiences from across different cultural groups. Data collection continued until the lead researcher was satisfied with *data sufficiency*, or the richness of the data generated from the interviews would lead to rigorous data analysis [22]. Unlike data saturation, which assumes that any further data collection will not produce additional added-value insights [23], data sufficiency recognises that “within a research paradigm that acknowledges both the uniqueness of human experience and the socially constructed nature of data—researchers can…continuously [dip] into a well of new understanding by iteratively revising interview guides, sampling new participants, and engaging in multiple rounds of data generation and analysis” [22]. The repetition of ideas and themes emerging across the data set suggested that the threshold for sufficiency was certainly met, if not the saturation threshold for this study’s specific data set.

Thematic analysis is a method for identifying themes and patterns of meaning across a dataset in relation to a research question [24]. A six-step thematic analysis qualitative framework developed by Braun and Clarke guided data analysis [25], setting up a rigorous process that led to *analytical sufficiency*, where the research team was confident the collective analysis broadly reflected the data provided by key informants [22, 26]. The first investigator reviewed a small selection of transcripts for early themes and concepts, from which the preliminary coding scheme was constructed. Then a second investigator coded all transcripts, revising the scheme iteratively to reflect emergent themes from interview responses. Qualitative data analysis software, QSR International’s NVivo 12, was used to code all transcripts, categorise the data and facilitate analysis across participant views. To ensure coding consistency, a third investigator independently coded at least 20% of randomly selected interviews. The investigators convened to share their categories; any discrepancies were resolved through discussion and negotiated consensus. Data analyses and interpretation were iterative, and all investigators participated in this process to identify and agree upon emergent themes and discuss their face validity. Two of the seven team members identify as coming from CaLD communities, and their input was invaluable in ensuring relevancy to broader CaLD communities. An overview of the research team, study design and analysis is described in S1 Appendix using the COREQ reporting format [27].

## Results

Twenty-nine interviews were undertaken with community and religious leaders based in NSW, Australia. These participants represented CALD communities originally from Asian, South Asian, Middle Eastern and African backgrounds. There were eight themes identified from analysing community leader’s perspectives on government communication strategies and processes with multicultural communities during the COVID-19 pandemic: 1) *“They know nothing”*: understanding the language and generational divide affecting CaLD populations; 2) *“I expected a lot more engagement”*: critically assessing the government’s engagement with CaLD communities; 3) *“How much perfection do we need*?*”*: expressing support for the government’s engagement with the CaLD community; 4) *“Bridging the gap”*: CaLD communities leaders stepping up; 5) *“With some translation*, *I would cringe”*: quality and scope of translations matter; 6) *“Most of them are tuning into overseas stations”*: relying on overseas health messaging among CaLD communities in Australia; 7) *“You can’t reach them by the traditional way”*: harnessing social media; and 8) “*The people want to know the truth”*: challenges of communicating complex medical information to CaLD communities. These themes are described in detail below.

### 1. “They know nothing”: Understanding the language and generational divide that impacts CaLD populations

The diversity and heterogeneity of CaLD communities present unique challenges in ensuring accurate public health messaging is accessible for all. One of the community leaders stated: *“Within the non-English speaking communities*, *there are some people that require more support than others”* [Community Leader (CL) 06]. Two of the most notable challenges highlighted by community leaders who participated in this research were: 1) an English language divide, where basic English proficiency was not sufficient–or absent—to fully understand the rapidly evolving public health messaging, and 2) a generational divide, where accessibility to quality translations of public health information was limited, especially in the absence of internet use and social media proficiency.

Key informants noted that some CaLD community members have basic English language proficiency, though many depend on community language newspapers or radio stations rather than mainstream English-language news outlets. *“The majority of migrants [only] have basic English language skills*, *so they need a lot of support*. *They need the information translated… to read and understand it well”* [CL 21]. Another community leader noted, *“They stick to their own people… They’re quite happy with their language among their own people*. *It’s easier for them to receive the information in [their community language] rather than in English”* [CL 16]. Furthermore, it was reported that some people from CaLD backgrounds do not have high levels of education and may even be illiterate in their own language and require complex public health information to be distilled into more simple and clear instructions.

Ageing populations within CaLD communities were also raised as a major factor in considering how to disseminate public health information. A digital divide may impact older CaLD community members, who are unable to confidently navigate the internet to access urgent critical public health information despite some community training efforts. While some older adults from CaLD backgrounds have family in Australia able to offer support, others are isolated and have limited outreach avenues. *“How much do [older people from CaLD backgrounds] know about what’s happening*?…*[W]hen I would go for walks in my [neighbourhood]*, *I’ve been curious to ask [older] people*, *what do they know*? *They know nothing”* [CL 17]. Community leaders emphasised the importance of public health messaging catering to the specific and unique needs of elderly CaLD community members.

Older people living in historically insular communities where CaLD migrants moved to areas for manufacturing industry jobs nearly half a century ago also warrant special consideration in relation to their English proficiency. The emergent large migrant communities meant immersing themselves in English was less necessary. *“There was no need for them to know any English; they would get a job and work for decades without learning too much English*. *Because there were so many of them*, *they didn’t need to learn English”* [CL 11]. However, as the population has aged, accessing information has become more challenging, relying on overseas details in their community language, which may not bear much use in the Australian context. The intersection of how people from CaLD backgrounds, English translations and interpretation, generational differences, the role of the internet, and technical public health information played out during the pandemic in Australia will be explored in more detail in the themes that follow.

### 2. “I expected a lot more engagement”: Critically assessing the government’s outreach to CaLD communities

Feedback from community leaders was largely critical of the government’s initial COVID-19 engagement and communication efforts with CaLD communities. There was a general sense of frustration with how essential public health information was disseminated to CaLD communities, with many community leaders pointing to limited resources that were translated into languages other than English, poor quality translations (see the third theme below on ‘Quality of translations matter’), and lack of outreach to the community organisations. One community leader noted, *“There were no information reaching the multicultural community*, *especially the non-English speaking backgrounds”* [CL 06]. Another observed that information about vaccines was limited. *“I did not see much engagement with the community about the vaccination*, *I must say…I [would have] expected a lot more engagement; a lot more”* [CL 17]. This respondent pointed to the daily TV news updates on the unfolding COVID-19 situation as the most notable engagement with communities. However, such outreach would only capture a section of the community with higher levels of English proficiency.

Participants acknowledged that while the beginning of the pandemic was worse regarding accessing information, communication did improve as the pandemic unfurled and new learning emerged. *“[Information] wasn’t that great at the beginning*. *There was a lot of individual work in the community to convey the message*. *The government wasn’t doing much in that regard”* [CL 21]. Though participants conceded that the availability of information in community languages slowly improved, there remained an information gap filled by community members stepping in to translate critical messages, such as general public health statistics, lockdown rules, and other relevant updates. *“I knew a few people who are doing it themselves*, *without pay*, *without anything*, *[with] nothing*, *just the individual—they were volunteering to do it”* [CL 21] (see theme four below on ‘Bridging the gap’). While communication with CaLD communities was enhanced as the pandemic progressed, several participants cited a lack of a coherent strategy targeting the diverse CaLD populations and an over-reliance on community organisations to lead in the delivery of public health messages in different languages throughout the pandemic.

### 3. “How much perfection do we need?”: Expressing support for the government’s engagement with the CaLD community

Criticism or frustration at the government’s outreach efforts was not shared universally across the key informants. Some community leaders expressed satisfaction with how public health messaging on COVID-19 and translated materials were distributed to CaLD communities. *“We are fairly satisfied*. *Whatever messages we get*, *we share with our community through our [online newspaper] …[W]e [also] motivate the people in our community to organise [through] Zoom meeting[s]”* [CL 02]. Online forum meetings were highlighted as essential for dispersed community members across the country to come together and share information regarding COVID-19. Similarly, others less critical of the government’s response suggested there needs to be limits on the number of languages the government is expected to translate information into. *“I just don’t understand how many languages we need to translate and how much perfection we need*. *It’s an emergency situation”* [CL 01]. This respondent also noted that compared to other places in the world, the ability of the government to cater to a wide range of languages should be commended: *“Somebody came up with so many translated documents*… *It was the best thing that has happened”* [CL 01].

Other respondents suggested that ensuring translated documents are created and distributed must be a shared responsibility between the government and the community and that the community has a role to play in facilitating knowledge transfer. Such cooperation may include volunteering to translate documents on behalf of the government. *“Many times*, *we have taken those documents and got a person to translate it…and then distribute it*. *What’s the big deal about it*? *I can…sit down for half an hour and [translate] it”* [CL 01]. However, while some respondents expressed ambivalence about translating certain documents, others took issue with the amount of time and skill required to do this work without funding or support.

### 4. “Bridging the gap”: CaLD community leaders and organisations stepping up

Several community leaders used the phrase ‘bridging the gap’ or ‘filling the gap’ when describing their organisation’s role in disseminating public health messaging to their relevant communities. Several participants noted that translating material and determining which networks to circulate the information was time-consuming, especially for organisations where funding was not available for formal translations. Leaders representing communities from South Sudan, China, Indonesia, and Iraq, among others, shouldered extra tasks to ensure that the government’s COVID-19 information reached their respective communities. *“There was a lot of individual work [in] the community to convey the message*. *The government wasn’t doing much in that regard*. *Only now with the second lockdown*, *we’re starting to see more information in community languages”* [CL 21]. Some of these community leaders translated and interpreted themselves, using their bilingual or multilingual fluency to ensure messaging reached the most vulnerable populations. In contrast, others engaged colleagues and international students to assist in translations and interpretations.

At the start of the pandemic, some community leaders conducted outreach to their communities by creating social media pages and video and radio content in their community language and sharing them online. The messages in the videos contained basic public health information for avoiding the virus, ranging from how to properly wash hands to social distancing etiquette. While the radio segments were broadcast to the broader population, the videos were distributed through a network of smaller organised groups, such as women’s groups. Another leader created a CaLD-community specific COVID Emergency Facebook page where awareness-raising and information sharing was accessible amongst community members.

While several community leaders stepped up to ensure public health communication had a further reach, many also acknowledged that this engagement came with a significant time and resource cost to the individual and the organisation (see the second theme above on government outreach). One leader noted that they received several phone calls a day from community members requesting clarification on several COVID-related issues, such as vaccines and lockdown rules.

*“Our community start[ed] to ask more questions*… *I receive[d] personally more than—I’m not exaggerating—50 to 80 calls daily*, *people asking me to clarify a few things*, *which I’m ready to do*. *I’m happy to [give people] the facts”* [CL 20].

Fielding phone calls, translating documents, sending messages, and uploading original videos were among the many modes of communication information intermediaries initiated to share pandemic-related public health information.

The suite of communication strategies employed by information intermediaries was not without its challenges. For example, when preparing for a radio segment, airtime is minimal, necessitating that complex public health content be distilled to a concise five-minute or less timeframe. Another leader noted that trawling through all public health data and information to determine the most pressing messages requiring translation and which dissemination channels to use was time-consuming. Similarly, reviewing the onslaught of COVID-related messages on the WhatsApp platform was reported as tedious, and community organisations needed support to navigate the information. *“I feel that if we can have people who looked through [the material] and see what’s relevant and convey that information to our communities*, *I think that would work”* [CL 03]. Such efforts took preparation and translation time of the respective community leaders, which was not necessarily reimbursed or part of their roles. However, most community leaders indicated they were prepared to shoulder such work given the extraordinary circumstances of the pandemic and the desire to protect their communities.

Some community leaders pointed to a missed opportunity to forge better partnerships between governments and community and faith-based organisations in engaging with CaLD communities during the pandemic.

*“When someone [well known] from the community and the government sit down together*, *they can talk*. *I believe this is how they can deliver [public health messaging] easier*. *This is what we’re trying to do… we complete*, *a little bit*, *the gap*, *in communicating real information with our community”* [CL 20].

A more collaborative approach between governments and CaLD communities, expanded here to include media organisations, was echoed by other leaders.

*“I think it’s a joint responsibility*. *The Health Department*, *with all the experts*, *has to provide the information and rationalisation*, *and then the main media outlet*, *like newspapers and television and other things*, *as well as the media targeting the multicultural community*. *I’m sure also that the ethnic or multicultural organisations have a bigger role to play once they are supported and given the right information and tools to spread the information”* [CL 19].

Community leaders were overwhelmingly united in the notion of shared responsibility and willing to ‘bridge the gap’ to ensure their communities were informed and supported throughout the pandemic. However, they also conveyed strong opinions that governments needed to bolster support to community organisations where governments did not have sufficient reach.

### 5. “With some translation, I would cringe”: Quality and scope of translations matter

Community leaders emphasised the importance of having high-quality translated material to reach the large swaths of their community where English is neither spoken nor proficient. *“It’s no use saying it in English”* was the response of one community leader when highlighting the critical need for more translated material [CL 15]. Furthermore, participants raised several important points related to the scope of the translations (determining which languages public health information should be translated), the nuances within languages and how critical it is to consider the wide range of dialects. Finally, several community leaders highlighted challenges and opportunities related to translating public health messages, whether through accredited bilingual community educators or through more informal mechanisms led by community organisations.

#### The scope of translations

One of the most critical decisions governments make about communicating to CaLD communities is the languages in which outreach materials are translated. Governments may prioritise translations based on census data indicating the highest proportions of languages spoken in the home. However, community leaders noted that some groups felt ostracised when their language was not part of the official list of translated languages.

“There have been other groups that have felt that they’ve slipped in between the cracks, and it was that whole premise of do you translate resources into the languages where there is the vast amount of people? Or do you go for those languages where maybe there are groups who are at heightened risk or have particular needs?” [CL 09].

Participants signaled that smaller CaLD communities may be at greater risk of pandemic-related harms because of the lack of community infrastructure around them.

As noted in the first theme above, translated material needs to take into account the educational background of their target audiences. *“You’re not translating to people who are educated and work…You have to be very clear [when you translate]”* [CL 04]. In some cases, translators and interpreters had to deviate from exact translations to make new concepts understandable, such as the use of QR (Quick Response) codes to check-in to venues. *“If I have difficulties understanding what they’re talking about because they’re using technical language*, *the jargon and all that*. *What about the common person*?…*Whether it’s COVID or non-COVID*, *this is the problem”* [CL 15]. Participants emphasised that the technical language associated with some public health information may be challenging for the average person, especially those who are not proficient in English.

Another important translation issue that community leaders consistently raised was the need for greater attention to the dialects *within* languages. Translations tend to be in the dominant languages of established CaLD communities. However, several community leaders advocated for additional resources to reach more diverse language groups, particularly in Indian and Middle- Eastern communities. As one community leader noted, even within seemingly cohesive CaLD communities, there is internal diversity among the dialects. A community leader representing the Indian community in Sydney said: *“We’re such a large community*, *and we speak so many different languages…that is the point I want to stress”* [CL 03]. This participant reported that most translated resources are in Hindi, Tamil or Punjabi. However, they continued:

*“[t]here are seven other languages that are spoken [in the Indian diaspora in Australia] which are not addressed… Although it is widely spoken here*, *not everybody understands Hindi*. *That’s where we step in because we can speak other languages*. *We use our ambassadors to speak*, *for example*, *in Gujarati*, *in Bengali…We use them as ambassadors to communicate the information in their native language”* [CL 03].

This community organisation filled a gap where additional translation and interpretation services were needed to communicate to community members speaking minority languages or dialects.

This sentiment was echoed by an Arabic-speaking community leader, who reinforced the importance of knowing which dialect would suit the audience.

*“For example*, *[i]n the Arab world*, *we have 18 countries…If we’re reading a book*, *it’s all the same language…*, *but the [spoken] dialect is very different… When I speak to a group of Iraqis*, *Sudanese*, *Lebanese*, *Tunisians*, *Kuwaitis or people from Dubai*, *I have to modify my language*. *It’s not the same dialect…[D]ue to the needs within the Arabic community*, *you’ll need to adjust your language”* [CL 04].

Another community member reflected on this observation: *“The problem is you cannot just translate an English text into an Arabic text*. *It doesn’t reflect the message’s exact meaning or essence”* [CL 19]. Other concurring key informants also noted that only educated members of the Arabic community could read documents translated into Arabic.

*“I’m finding that if we only rely on the translation of documents*, *[it’s] a problem because it’s tough to translate documents into plain Arabic*, *especially when we don’t even have it in plain English*. *In the Arabic language*, *if anyone from any of the Arab countries is to read it*, *that person has to be educated”* [CL 15].

Challenges with accuracies *within* languages played out in Chinese communities as well. One community leader noted that most government communication on COVID-related health information was translated into simplified Chinese, the written language of mainland China that emerged in the mid-twentieth century, to facilitate the more complicated traditional Chinese characters and promote literacy nationally. However, people from Hong Kong, Macau, and Taiwan, for example, do not necessarily read simplified Chinese; they continue to read traditional Chinese. Furthermore, some migrants from Hong Kong may not be proficient in English despite the historical British colonial rule. One community leader shared an example from a few years past when a local government council wanted information translated into simplified Chinese. When this community leader pushed back that some migrants only read traditional Chinese, the council responded: *“‘Because the statistics show that the majority of the Chinese are from Mainland China*, *they go for simplified Chinese*.*’ Of course*, *as a member of a small-sized community*, *I can’t say too much…[but] I wanted to speak out on behalf of these people”* [CL 09]. While this community leader later acknowledged improvements were made during the pandemic, they did caution that checking translations for accuracy and the appropriateness of the translated language should remain a priority.

#### The process of translating public health material

Early in the pandemic, one community leader noted that translating COVID-19 health advice was too slow. *“There was no information reaching the multicultural community*, *especially the non-English speaking backgrounds”* [CL 06]. Translation procedures could not keep up with the fast-paced shifts in messaging as the pandemic evolved, sometimes leaving days between a change in health advice and an official translation being circulated, at which point some advice was already outdated. *“I think [it] took too long to communicate with the multicultural communities and to get our leaders involved… and [now] we have to be very quick and fast”* [CL 15]. Efforts were undertaken to accelerate the translation and outreach processes as the pandemic progressed, both by governments, primarily through bilingual community educators, and community organisations (see the fourth theme above on ‘bridging the gap’).

The bilingual community educators, whom Local Health Districts (e.g. community public health units) within the NSW health department regularly engage, were seen as an asset in bridging the government’s health advice with the community, providing an essential service for communicating health advice to the broader community.

*“[Bilingual community educators are] great because they can speak the languages and communicate the information… They’re trained*, *so [their services] are very reliable and accurate*. *I think it’s good to employ bilingual community educators because the language proficiency may not be as good across the community”* [CL 03].

This community leader noted that bilingual educators–as well as general practitioners (GPs)–are often tapped to translate written resources for community organisations or speak to the media, and some feel these professionals should be utilised more for government translation and interpretation. *“Why not use GPs to communicate this information*? *We use them*. *We are often approached by the media*, *for example*, *radio*, *wanting to interview a GP*. *We connect them with GPs who speak other languages”* [CL 03]. The expanded role of informal information intermediaries is highlighted as a potentially important component in future pandemic preparedness with CaLD communities.

One community leader emphasised the need for government to reinvest in community engagement, in knowing one’s population, rather than rely on formal modes of communication. There may be missed opportunities to work with the people on the ground to disseminate information rather than being over-reliant on hard copy translations. Print material was especially flagged as being less useful. This participant said, *“People don’t read”* [CL 03]. While this is likely an overstatement, it does raise the possibility that other modes of communication should be foregrounded–social media in particular–to reach a wider swath of the CaLD communities (see the seventh theme below on harnessing social media). It further emphasises the need to focus on short, sharp pieces of information rather than expect community members to read through detailed health advice. Participants noted that over-circulating print material, or long-form electronic details, had the opposite effect of its intent. It was reported the ‘bombardment’ of health information emails led to people ignoring them rather than engaging with them.

Another community leader highlighted the value of bilingual community educators beyond the simple act of translating. Their intimate knowledge of the community and culture allows for a more nuanced translation that may consider people’s understanding of Australian culture and English language ability, education level, and years they have lived in Australia. There is also an undercurrent of trust between the bilingual community educators and the wider community that does not animate well from a generic translated document. One participant noted that while people from CaLD communities may have a basic understand of English, they ‘absorb’ more in their community language.

Where bilingual community educators could not fill the knowledge gap with CaLD communities, as noted in theme four above, community organisations stepped in. Community leaders discussed how the translation process unfolded within their organisations, including, in some cases, the leader taking the initiative to translate and disseminate COVID-19 information. In other scenarios, there was a more thorough system of checks and balances, where the translator, editor and proofreader were all separate individuals. *“Most of the material we translated [was] perfect because it will go through different channels*. *The person translating is not the same person proofreading it”* [CL 04]. For some CaLD organisations, there was a further layer of cultural or religious sensitivity checks. While perhaps less relevant for COVID-19, it is essential for other health concerns, such as sexually transmitted infections.

Community leaders expressed frustration at the quality of translations they saw. *“In shopping centres*, *for example*, *I would pass by a [notice] board with some translation and sometimes cringe because of the language*. *I’m not sure if they use Google translate…”* [CL 04]. Another community leader commented similarly: *“I did see that there has been some improvement [in the translated materials] over the year*, *but I just hope that they will also have somebody to [double] check to see that it is the way it should be translated”* [CL 09]. While these two community leaders did not specify if the translated materials were government-endorsed translations, it does point to an unease with the accuracy of public health information translations more generally. While there has been criticism about the quality of translated material, anecdotally, people have also pointed to the original messages in English as being less precise, further challenging translation efforts. However, in raising these concerns, another tension arose among our participants: whether translators and interpreters should act only if certified by the National Accreditation Authority for Translators and Interpreters (NAATI). Assessment fees for individuals and institutions can range from hundreds to thousands of dollars, respectively, and do not include the additional course fees, which reportedly cost upwards of A$2000 [20]. Two community members noted that despite experience in translating and interpretation, they do not have a license for translation because they cannot afford the fees. *“We just speak the language and make sure it’s right for the public”* [CL 04]. A clear message from community leaders was that they grappled with ensuring translation and interpreting services could most rapidly and accurately reach their populations through formal and informal means.

### 6. “Most of them are tuning into overseas stations”: Relying on overseas health messaging among CaLD communities in Australia

Reliance on accessing COVID-19-related health information from overseas was a dominant theme among our community leaders. While some information of a general nature about COVID-19 –what is the virus, how does it spread, how to wash hands and socially distance—in community languages was helpful, that heavy dependence on information from overseas overshadowed locally relevant information about lockdown rules and government support, for example, leaving some CaLD community members in the dark about how COVID-19 was playing out in Australia. Representatives of Serbian, Middle Eastern and Indonesian communities, for example, expressed concern that elderly members of their communities were digesting COVID-19 information exclusively from overseas resources. Older generations of CaLD communities may access their news from overseas newspapers and streaming TV services, missing critical local information. *“There wasn’t proper communication*, *I have to say*, *from an Australian perspective; what is happening here*, *what is available here*? *That’s the reality for those elderly [people]”* [CL 11]. One community leader noted that as CaLD populations age, coupled with the heavier reliance on the internet and social media, some local community language newspapers have declined circulation. As a result, they attract fewer advertising dollars, and the commercial viability of these news outlets is compromised, cutting off an important source of local information for elderly migrant community members, hence their pivot to overseas resources.

Accessing overseas information is not limited to just older people from CaLD backgrounds. Newly arrived migrants and refugees also needed better support to access information in Australia. *“The government needed to collaborate with community workers for this message to reach the community…When new migrants come here*, *they still watch the news in their language…whatever they used to watch overseas”* [CL 21]. Access to overseas media also continues to occur amongst the general CaLD population.

“*Talking about the Arabic community*, *you’ll find that most are tuning into overseas stations*. *We may have this problem because the perception of COVID-19 is so different in Arab countries*, *and we will never know the truth of the numbers…If we say one thing and they’re watching another*, *the overseas channel*, *they get another message*. *It’s a conflicting situation for them”* [CL 15].

In the context of a pandemic, bypassing local mainstream media in favour of overseas media meant critical public health information was not easily accessed by some members of the CaLD communities, both young and old.

Community leaders acknowledged that they were actively seeking health information from overseas governments, given material was already in the community language.

*“Because I speak Arabic*, *I can access other Arabic countries’ media*, *and I found some material from [an overseas] Health Department*. *I sent it to [a multicultural organisation]*, *and other people are saying ‘use it’ as a model of addressing the issue in Arabic*. *There is no need to reinvent the wheel if somebody did something good”* [CL 19].

At the same time, some community leaders were cautious about accessing overseas information, fearing it would not be relevant to the Australian context.

*“I access the translated version when I need to translate it*, *for example*, *or to proofread it within my role…but normally the fastest thing is to access whatever you have*. *It’s always in English because the Arabic one could relate to the Middle East…I don’t want to give the wrong information while reading something unrelated to Australia”* [CL 04].

The concerns about CaLD community members accessing COVID-19 related health information overseas were not universally shared amongst our community leader interviewees. One participant acknowledged that while some people may not understand English, the vast majority were accessing information locally, primarily through the daily State government press conferences during the height of the lockdowns. *“Honestly*, *I don’t access any overseas information…I wouldn’t bother*. *What for*? *Why would they get information from overseas*? *Why can’t they get it here*? *Service New South Wales*, *if you can download their app for COVID*, *the information was there”* [CL 07]. While there may have been deviating opinions on the overall impact of accessing overseas public health information, community leaders shared that some of their members relied on pandemic messaging from overseas when local public health messaging is inaccessible, which sometimes contradicted or misaligned with the government’s advice.

### 7. “You can’t reach them by the traditional way”: Harnessing social media

There was a resounding agreement amongst the community leaders about the growing importance of harnessing social media in disseminating critical public health messaging. Mainstream media is no longer sufficient in reaching most population groups, especially CaLD communities.

“*You can’t reach them by the traditional way*. *If you want to reach them*, *you will use platforms like Facebook*, *Instagram*, *[messaging apps like] WhatsApp [Signal or WeChat]*, *whatever they use… If you want [to reach CaLD] communities*, *you’re going to have to use social media”* [CL 21].

As noted above, traditional modes of community engagement, such as through local newspapers, which may be produced in community languages, are slowly vanishing under the wave of social media, which lowers circulation numbers, leading to a disincentivizing of advertising revenue. During the pandemic lockdowns, community leaders had to shift face-to-face interactions to online platforms. Welfare and well-being checks would be conducted through text messaging and translated recordings by community leaders and organisations about symptoms of COVID-19 and where to get tested, for example, would circulate on messaging platforms. *“Everyone is so attached to their mobile phone*… *They can listen on WhatsApp and read through Facebook and [other social media platforms] like that… Social media will be the best way to reach them*, *from the young to the old”* [CL 16]. This community leader also noted that one must not rely on social media in isolation and that additional supplementary forms of communication, including flyers and radio, also have added value in reaching those without social media access.

Several community leaders used social media platforms and web-based messaging to receive information, filter it and forward the most important and relevant health information to various community groups. “*I receive information from various organisations*, *government and non-governmental organisations*, *and the senior services agency*. *I also share with them to my Facebook group so that people are on top of other things”* [CL 09]. One community leader also noted that social media videos contain essential information in one’s language and dialect, and links can easily be forwarded to community members and should be further leveraged by the government.

*“The Arabic translated messages from the government*, *from the Health Department*, *is not well-presented or well-distributed… I think [health messaging] should go to Facebook*… *They should use Facebook to send it to almost everybody or everywhere—something in Arabic so that people read it”* [CL 12].

Most community leaders were impressed by the importance of using diverse modes of social media to communicate public health information about the COVID-19 vaccine.

Community organisations were adapting to provide COVID-related information. In conjunction with a multicultural organisation in Western Sydney, one community group recorded several webinars in Arabic, covering a wide range of COVID-19 topics, such as self-isolation, improving immunity, treatment and vaccination. These webinars typically featured eminent community-based doctors and health specialists to disseminate key COVID-19 information to the Arabic-speaking population of Western Sydney. These webinars were then recorded and posted on social media, such as YouTube and Facebook, and circulated, increasing their visibility across the community.

While some community leaders expressed excitement at the possibilities social media brings in disseminating information, concerns were also raised by others related to the theme above–on ‘bridging the gap’—about the volume of the messages circulating and resources community organisations and individuals needed to sift through those messages to find the most relevant ones to convey to the broader community. Furthermore, while there are clear benefits in shifting additional resources to increase the government’s social media engagement, there are trade-offs with these platforms, particularly the proliferation of false and misleading information.

*“People rely on social media*, *which sells all sorts of stories*. *We had to take it into our own hands*, *actually do the work and talk the talk [to inform our community]*. *We did not see much from the government [engagement with] the community”* [CL 17].

Some disagreed on whether the government’s outreach to CaLD communities was acceptable. One participant noted that outreach was absent, primarily through social media. *“I haven’t received one message about COVID-19 and vaccines [in my community language]”* [CL 21]. Other participants, however, expressed satisfaction with the outreach and communication strategies employed by the government, remarking that the amount of pandemic-related information available across several platforms has been sufficient. *“Everywhere you go*, *information is there… Published*, *posted*, *I’m seeing [information] everywhere on social media*… *I appreciate all these strategies”* [CL 18]. However, this participant also cautioned against relying too heavily on print material. *“If you want to hide any information from [certain] people*, *put it in writing*, *that is a saying”* [CL 18]. Several other community leaders were critical of print documents as the dominant mode of disseminating information, echoing a blunt sentiment shared above that *“no one reads that”* [CL 11]. These community leaders suggest that seeking alternative delivery modes–such as through more targeted social media messaging—may be needed to reach those who avoid print material.

Where print and social media fail, the last resort is to engage in one-on-one contact in person or on the telephone. *“No one reads posters anymore*, *and no one reads general emails*. *You have to give me a call*… *I need to talk to a live person”* [CL 11]. Given the costs associated with engaging a person to reach individuals, this is the most resource-intensive mode of communication, losing the broad reach of other messaging. *“You don’t have the money for [hiring interpreters]*. *You have money for other stuff*. *Printing the poster’s much cheaper than paying a person to go somewhere”* [CL 11]. While community leaders are signaling that social media will need to play an increasingly significant role in disseminating critical public health information, more traditional modes of communication and engagement are still necessary to reach the diverse needs of CaLD community members, as well as to safeguard against the misuse of–and misinformation on—social media.

### 8. “People want to know the truth”: Challenges of communicating complex medical information to CaLD communities

The rapidly evolving public health information during the pandemic created unique challenges for outreach efforts to CaLD communities. Increasingly, the challenge of providing clear, scientifically based advice about COVID-19 vaccinations has emerged. The evolving data on the efficacy of the newly created COVID-19 vaccines, coupled with intense media scrutiny on the possible side effects and one’s own personal risk assessments, complicated the vaccine roll-out for the general population and CaLD communities alike. In discussing the challenges of communicating complex medical information to their community members, CaLD leaders noted they initially struggled to access information about the vaccine that their community members wanted. While some of the technical details on the vaccine were available, concerned community members had additional questions about the safety and efficacy of vaccines (e.g. Which vaccine is better? What are the side effects?). One community leader expressed disappointment in the public health communication, especially strategies to target multicultural communities.

*“I am a bit disappointed because I consider myself to be relatively highly educated*, *and I had [feedback from people] who had problems with understanding what we’re up to [in the pandemic]*, *or getting conflicting messages and thinking the government has been a bit slack in applying and implementing policies*, *and then changing their mind*. *If I’m finding that hard*, *and I do feel for the rest of the community*, *or for people who don’t have that certain level of exposure to education”* [CL 15].

While a broad understanding of the evolving nature of the pandemic was a challenge for most of the population, information about vaccines represented a flashpoint for misinformation.

One community leader emphasised that people want to be informed about the vaccine. *“The people really want to know the truth”* [CL 20]. While noting the government was initially not forthcoming with information about the vaccine targeting CaLD populations, community organisations *“tried to explain to our community the real fact of having the vaccine”* [CL 20]. However, feeling ill-equipped to answer some basic questions about health messaging, community members turned away from community organisations and towards other sources of information on the internet, at times unhinged from data or facts. The resulting conclusion led to community leaders fielding a significant amount of phone calls from concerned community members seeking more information (see CL 20 in the fourth theme above). Several community leaders have grappled with how to reduce the spread of false and misleading information about COVID-19 in general and the vaccine in particular. Some community leaders noted that CaLD community members–older generations in particular—carry scepticism of government information, a by-product of some migrant and refugee backgrounds previously living under the authoritarian rule where governments could not be trusted. However, while most young people in the community (20 years and younger) know English and can access information more easily, older, first-generation migrants and refugees, especially those born under dictatorships, *“don’t believe what the government says… Everybody’s suspicious of everything”* [CL 12]. Such distrust in the government adds another layer of complexity in reaching older people from CaLD backgrounds, on top of several challenges already highlighted above.

When it comes to COVID-19 vaccine information, community leaders had one key message: that vaccine information should come from well-known bilingual medical or health professionals from the community, who have the relevant public health expertise, and are credible sources of information. *“I believe that community leaders should not go and just pass on messages related to health… the only people that [pass on public health messages] efficiently are the community doctors themselves”* [CL 22]. Some leaders noted that while other community members, especially respected elders and faith-based leaders, can support these efforts, their core business is not health-related messaging and, as such, should be secondary–albeit important—sources of information for the community given their elevated status after health professionals lead the advocacy efforts. *“[A religious leader] does not have health expertise whereas the message is more credible if it comes from a health professional”* [CL 14]. There were also suggestions that some faith-based leaders may voice concerns about the vaccine and thus are not best suited to act as health promoters. *“[N]one of [the religious leaders] have been vaccinated…If they don’t believe [in the vaccine] themselves*, *how can they spread the message*?*”* [CL 14]. This community leader also noted that providing space for one-on-one consultations with a health practitioner, accompanied by an interpreter, could have helped to address their concerns about the vaccine. *“I still believe…bilingual health workers [should] deliver the messaging in their first language in the community”* [CL 14]. Another community member suggested simplifying the messaging. *“Not many people understand what this vaccine is*. *But they could understand it very easily by explaining in a simple way and in simple language”* [CL 16]. Community leaders also singled out approaching GPs from multicultural communities to promote the vaccine roll-out specifically.

*“[W]e should have perhaps started with some multicultural*, *ethnic background GPs to get them on board*, *to say to people they can go to them [for vaccine information]*. *It has been very*, *very vague messaging*. *No*, *I think this is one thing that we missed”* [CL 15].

Community leaders suggested that CaLD health professionals could strike the right balance of explaining public health information scientifically, but not too technically, while providing clear, straightforward advice in accessible community languages [21].

## Discussion

The overwhelming message from community leaders was a sense of shared responsibility between their organisations and the government in communicating pertinent and accurate COVID-19 related information to CaLD communities. Community leaders expressed a sense of duty to keep their community members safe. However, there were significant costs shouldered by community and religious leaders, other individuals and organisations related to resources and time that need to be acknowledged by governments in the future in preparing for future disease outbreaks. Further public health information campaigns that target CaLD communities have several considerations for improving their translating and interpreting capacity. Several ideas are emerging from discussions with community and faith-based leaders–the information intermediaries—that governments should consider: 1) improving communication between governments and CaLD organisations; 2) responding to the specific CaLD needs with greater agility; 3) foregrounding social media in public health communication strategies; 4) reinvesting in local public health units to know their population; 5) developing a health ambassadors model program; 6) preparing a hybrid model of translators/interpreters to fill the gap; and, 7) reimagining vaccine information campaigns to better target CaLD communities.

### Improving communication between governments and CaLD organisations

Recent literature has reported the communication gap between CaLD members and the public health dissemination system, caused mainly by a lack of access to COVID-19 specific information and ineffective communication channels for CaLD groups and communities to receive necessary information [3, 12, 18, 19, 28–31]. This was particularly problematic for those with low digital or English literacy levels, and older ethic minority adults [18, 32]. A similar tension emerged from our discussion with community leaders, suggesting governments placed significant responsibility on community leaders and organisations to deliver public health messaging to communities. To address the discord between governments and the community, one leader suggested more communication between government representatives and community leaders is required to forge a more collaborative approach between governments and CaLD communities. The government expected community leaders to disseminate key public health messages, and the vast majority were willing partners in the efforts to inform their people; however, while there was a shared responsibility, there was not always a shared cost. While community leaders were prepared to ‘bridge the gap’, appropriate funding and support for costs incurred for the significant time and resources required was an issue repeatedly flagged by the participants. With additional government support to bridge important gaps in the pandemic response, community leaders believed that multicultural organisations had a more significant role to play in educating their communities and circulating relevant public health information.

### Responding to the specific CaLD needs with greater agility

Agility is not a term typically used to describe government action. However, based on the general lesson learned about the pandemic [33] and insights from the community leaders throughout this paper, it is clear that with pandemics of the nature and scale of COVID-19, the ability of the government to rapidly shift and pivot based on the current data is required for an effective public health response. A vital aspect of this response is having effective mechanisms to engage and communicate effectively with the community, especially those from CaLD communities where English proficiency is limited and trust in governments is strained [31, 34, 35].

It was evident in the early stages of the pandemic that the public was not prepared for the evolving nature of public health advice (e.g. not wearing masks at the beginning of the pandemic to safeguard stockpiles for health care workers and patients, to recommending mask wearing by all members of the public when outside one’s home) [36, 37]. In future pandemics, governments need to make clear at the outset that public health advice is not static and will evolve and change as new data emerges and resources change. In doing so, the community can be prepared for shifting advice daily when necessary. Alerting CaLD communities, especially those with low English language proficiency and less community infrastructure, and the community organisations that serve them about the possibility of evolving public health advice will be an essential feature in future pandemic preparedness.

### Foregrounding social media in public health communication strategies

One of the strong messages from the community leaders is that a suite of communication strategies is needed to reflect the diversity of CaLD communities, the generational differences in accessing internet-related information, and those with low English proficiency. While public health messaging through TV, radio, newspaper, and posters must remain among the modes of communication, it is clear that CaLD communities have increasingly engaged with various social media platforms to access and share pandemic-related information. Social media platforms such as Facebook, YouTube, and WhatsApp were widely cited by our participants as popular in their communities. The rapid availability of public health messages circulating through these platforms was critical to informing the CaLD communities about the pandemic. However, there were several pitfalls cited by the community leaders in using social media. First, the source of content was an important consideration. As noted in the theme above, ‘Bridging the gap, ’ community members readily stepped in to translate and interpret critical public health information initially inaccessible to CaLD communities and posted these resources on various social media sites. The organisations often did so voluntarily, though this additional engagement strained many organisations’ resources. Second, access to social media meant the general public, including CaLD communities, had easy access to a mirage of false and misleading COVID-19 information. The susceptibility to incorrect information led to further strains within community organisations to field additional questions about the pandemic. It also led to concerns about vaccine hesitancy and challenges getting the general public on board for mass COVID-19 vaccine campaigns. Finally, another challenge with social media was the ease of accessibility to overseas pandemic information that may not have applied to the Australian context. While some CaLD communities may have tuned into overseas TV and radio programs, the rapid exchange of overseas information in their community language coloured their perception of the pandemic locally. Awareness of the potential reliance on overseas information, which may or may not be relevant to the local context, needs to be a consideration of pandemic preparedness planning with CaLD communities going forward.

Furthermore, reviewing and revising public health messaging strategies will need to place greater attention on the strength of engaging with social media, while also acknowledging the pitfalls of the platform and mitigating against negative effects [38]. At the same time, as generations evolve, there may need to be a sunsetting of more traditional modes of communication–translated newspapers and radio programs, for example–in favour of internet-based communication strategies. While it is clear that harnessing social media to disseminate critical public health information will play a significant part in future communication strategies, it will not be the *only* part of a strategy. A variety of communication platforms with substantial reach and one-to-one contact will still be required in the years to come.

### Rinvesting in local public health units to know their population

One of the important initial lessons from the pandemic in Australia was that early and sustained investment in local public health units made a material difference in how the pandemic played out in real-time, particularly when comparing COVID-19 incidence rates and distribution in NSW and Victoria (the second most populated state in Australia) [39, 40]. Prior to the pandemic, NSW’s public health approach was more decentralised, where local public health units had significant command of their populations. By contrast, the Victorian public health approach was centralised, leaving public servants based in Melbourne to make decisions across the state, where knowledge of the local population and area may have been limited. This dichotomy in public health approaches across State/Territory lines was a critical difference in the initial trajectory of the pandemic [41]. Greater research on how the various local public health responses to CaLD communities, specifically across State/Territory lines, is warranted.

The CaLD community leaders emphasised the need for governments to reinvest in community engagement and in knowing one’s population [42]. This could take a variety of forms, including increased investment in local public health unit outreach with CaLD communities, formalising more partnerships with community organisations, fostering regular engagement with community organisations both during the pandemic and non-pandemic periods, and ensuring an obvious understanding of the cultural and linguistic diversity *within* the community so that no one gets left behind in public health emergency communication strategies. Such investments should not rest solely on the shoulders of public health but have broader appeal to other emergencies, such as fires and floods, where rapid transmission of urgent information is simply and communicated to the affected communities in a language they understand.

### Developing a health ambassadors model program

The community leaders suggested that public health information should come from health practitioners, who have the medical and public health knowledge, high levels of cultural sensitivity, and the trust and respect of the local community. Having called into question the possible role of community leaders and faith-based leaders not specialised in medicine or public health could play as spokespersons in vaccine information dissemination strategies, community leaders strongly endorsed a model of more personalised engagement between community members and health practitioners. Some community leaders expressly stated that investment in one-on-one engagement is preferable, even if not financially practical. While resource-intensive, governments could consider a variation of a model used in Victoria to support vaccine uptake, called Vaccine Ambassadors [30]. These ambassadors were trained to travel door to door to engage with local community members and have conversations about the vaccine, answering questions about safety and efficacy and where to access vaccination clinics. Further research on the impact of these Vaccine Ambassadors and the potential to broaden their scope to ‘health ambassadors’ could be a way forward to bridging government-led public health with CaLD communities.

### Preparing a hybrid model of translators/interpreters to fill the gap

The volume and urgency of public health information disseminated to the public forced community leaders and organisations to step in to fill the gap in reaching CaLD communities. In doing so, many community leaders leveraged their connections to the community and their language abilities to translate and interpret the public health messaging. However, some acknowledged that they did not have the official NAATI accreditation, which recognises trained translators and interpreters. A tension emerged between the need for accurate translations and the urgency for information to be disseminated. There were concerns that individuals voluntarily undertaking translations themselves could misinterpret data, misrepresent information, or inadvertently change meanings, signalling the engagement of NAATI-certified translators would be necessary to ensure the accuracy of the translation. There may be an opportunity for a hybrid model where governments can call on NAATI-accredited translators and interpreters, but also informal translators networks, to disseminate critical health information. It was suggested to have someone from the affected community to cast an eye over an official translation to review it for accuracy and relevancy to the community. Finding the right balance of accuracy, speed, breadth of dialects, and cultural knowledge appeared to be a priority for the community leaders–and there needed to be a way forward to use both accredited and non-accredited translators during public health emergencies.

### Reimagining vaccine information campaigns to target CaLD communities better

One of the most important lessons from this COVID-19 pandemic is how to develop and implement vaccine information campaigns to reach CaLD communities more effectively [43, 44]. Understanding the effectiveness of public outreach to inform community members and support their decision-making process is critical to future public health emergencies. Community leaders emphasised the need for increased outreach to multicultural communities to support their decision-making about the vaccine. While there may not be one approach that will best help CaLD communities, offering them factual, scientifically based information that is understandable to the layperson is an essential component of any strategy. As noted above in the other themes, harnessing social media, printing flyers, and promoting multicultural health communication on radio and TV all need to be part of a multi-prong strategy of vaccine information outreach.

### Study strengths and limitations

While the interviews were undertaken in Australia, the findings have relevance to similar countries with diverse cultures, ethnicities, and languages and where health departments grapple with how best to respond to, engage with, and support CaLD populations. The findings and discussion are helpful for governments in identifying the elements to support change and to enhance future pandemic–and broader public emergency—responses, especially as they relate to communication strategies.

As with any interview study, the data we collected were limited by the willingness of people to share their views; in particular some community leader may have a general distrust of government and/or the academy (‘being researched on’, such as this study which was initiated at the university–along with CaLD colleagues, as opposed to ‘with’, such as with a true co-design process with community leaders at the outset). However, there were very candid and detailed reflections in many of the interviews providing important insights to the CaLD communities’ experiences for future consideration. The findings are also not representative, but rather include a range of perspectives to inform future pandemic responses for CaLD communities. Furthermore, the limitations of this study stem from the narrow geographical setting from where most participants were recruited–namely Western Sydney, NSW, Australia, which is the area of greatest diversity and CaLD representation in Sydney. Furthermore, all of the community leaders identified and recruited were adult males. It would be interesting to test some of the findings from this study with new research that focuses on the perspectives of other CaLD community leaders, such as female leaders, young leaders, ‘respected elders’, and other diverse representations to see how gender and age may or may not impact perspectives on public health communication strategies and engagement during the pandemic and lessons learned.

## Conclusion

Culturally and linguistically diverse communities are an important part of the Australian population, and it is imperative that their needs are met as part of the Australian response to ongoing and future pandemic responses. Unfortunately, evidence suggests that CaLD communities bore a significant burden in the Australian response to the COVID-19 pandemic. Recognising the outsized impact the COVID-19 pandemic had on CaLD communities, this study aimed to understand the perceptions of CaLD community and religious leaders, who have an active role in the delivery of services and other social support to CaLD communities in Australia, in regard to COVID-19 public health communication and engagement activities. The results signaled that the overwhelming message from community leaders was a sense of shared responsibility between their organisations and the government in communicating pertinent and accurate COVID-19 related information to CaLD communities. They expressed a sense of duty to keep their community members safe. While acknowledging this shared responsibility, community leaders and others shouldered significant costs related to resources and time that need to be acknowledged by governments in preparing for future disease outbreaks. They felt that governments needed to improve communication with CaLD organisations in order to more agilely respond to the specific needs of CaLD communities, and that information intermediaries can play a role in facilitating greater cooperation.

It is imperative of government, academia, health bodies, and community and faith-based organisations to heed the lessons learned and ensure that CaLD communities do not get left behind in future pandemic and public health emergencies. A critical component of ensuring an effective public health response sensitive to the lived experience of CaLD communities is to develop communication strategies that are reflective of the CaLD populations in terms of diversity, language ability, educational background, and internet knowledge. Information intermediaries play an important role in influencing the flow of communication, as well as shaping and informing their community’s reality and knowledge in a culturally appropriate and salient manner. Given the technical details about the COVID-19 virus conveyed in government information campaigns and the media, ensuring the most vulnerable populations, including people from CaLD backgrounds, access clear, concise and timely public health messaging from both governments and community organisations requires further attention and engagement with information intermediaries. Communication strategies that include a diverse, flexible and more agile approach to reaching CaLD community members should consider new modes of engagement beyond traditional translation and interpretation services. This can be facilitated by greater communication between governments and CaLD community organisations to discuss their shared responsibility and how it can be properly resourced for future pandemics, and public emergencies more broadly.

## Supporting information

S1 Appendix COREQ consolidated criteria for reporting qualitative research. (DOCX)
