# Supplementary material for: “It’s no use saying it in English”: A qualitative study exploring community leaders’ perceptions of the challenges and opportunities with translating and interpreting COVID-19 related public health messaging to reach ethnic minorities in Australia
Source: PLoS One. 2024 Feb 29;19(2):e0284000. doi: 10.1371/journal.pone.0284000 (PMC10903877; doi:10.1371/journal.pone.0284000)
Supplement: S1 Appendix — (DOCX) [file pone.0284000.s001.docx]

**S1 Appendix: COREQ Consolidated Criteria for Reporting Qualitative Research**

| **No** | **Item** | | **Description** |
| --- | --- | --- | --- |
| **Domain 1: Research Team and Reflexivity** | | | |
|  | **Personal Characteristics** | | |
| 1 | Interviewers | HS, BHR | |
| 2 | Credentials | The two interviewers between them hold Bachelor, Master and PhD qualifications in behavioural science, social work, social research, medicine, and public health. | |
| 3 | Occupation | Academic staff (HS, BHR, AH, IA, AM, LW, EW) and public health practitioner (LW) | |
| 4 | Gender identity | Female (HS, AH, AM, LW, EW), male (BHR, IA). Two participants identify as from CaLD backgrounds (IA, AM) | |
| 5 | Experience and Training | Experienced researchers and interviewers, have undertaken several qualitative studies, trained in interviewing and qualitative analysis. | |
|  | **Relationship with participants** | | |
| 6 | Relationship established | None of the interviewees were known to the interviewers prior to the interviews. | |
| 7 | Participant knowledge of the interviewer | The participants in this study were representatives of the various community and faith-based organisations from New South Wales, Australia. The interviewers were not known to participants before the interviews were conducted. | |
| 8 | Interviewer characteristics | Established researchers with experience in vaccination, public health, health services research, primary health care, and culturally diverse health research. Collectively the interviewers have more than 40 years of research experience. | |
| **Domain 2: Study Design** | | | |
|  | **Theoretical Framework** | | |
| 9 | Methodological orientation and theory | Exploratory | |
|  | **Participant Selection** | | |
| 10 | Sampling | Purposive and snowballing, with participants sought across Australia. Participants were also asked to identify other participants to be interviewed. | |
| 11 | Method of approach | Emails which included information on the study, participant information and consent forms. | |
| 12 | Sample size | 30-40 | |
| 13 | Non-participation | 11 participants didn’t respond. | |
|  | **Setting** | | |
| 14 | Setting of data collection | Conducted via online videoconference platform and telephone | |
| 15 | Presence of non-participants | No non-participants were present for the interviews | |
| 16 | Description of sample | Participants were representatives from CaLD community organisations and groups, including Asian, South Asian, Middle Eastern and African backgrounds, from New South Wales, Australia. | |
|  | **Data Collection** | | |
| 17 | Interview guide | Provided in advance upon request | |
| 18 | Repeat interviews | No | |
| 19 | Audio/visual recording | Audio recording | |
| 20 | Field notes | Limited reflective notes were captured following interviews. These notes were not included in the qualitative analysis. | |
| 21 | Duration | Ranged between 20 minutes to 60 minutes | |
| 22 | Data saturation | Purposeful sampling to ensure range of experiences from across different cultural groups were captured and described. As such sampling continued past basic conceptual/thematic sufficiency, if not saturation, to ensure a range of cultural perspectives were included. | |
| 23 | Transcripts returned | No | |
| **Domain 3: Analysis and Findings** | | | |
|  | **Data Analysis** | | |
| 24 | Number of data coders | Initial coding was done by HS and EW. BHR contributed to development and refining concepts identified. Codes, concepts and higher order themes were discussed by all authors. | |
| 25 | Description of the coding tree | No, not necessary given the focus of the analysis is largely descriptive rather than being theory-driven. | |
| 26 | Derivation of themes | Codes and concepts were derived through open coding of interview transcripts, these were then tested and consolidated into higher order themes. | |
| 27 | Software | NVivo 12 | |
| 28 | Participant checking | No | |
|  | **Reporting** | | |
| 29 | Quotations presented | Yes, selectively to illustrate findings | |
| 30 | Data and findings consistent | Yes | |
| 31 | Clarity of major themes | Yes | |
| 32 | Clarity of minor themes | No, the focus in this paper is on major analytic findings and higher-order themes, given the focus of the study. | |
